# Community Mental Health in Postgraduate Education of Psychiatrists and Public Health Professionals- A Proposal for a New Subspeciality

**DOI:** 10.1192/j.eurpsy.2024.393

**Published:** 2024-08-27

**Authors:** I. Polat, O. Karadag, A. Tiryakioglu Engin, M. Altıner Yas, B. O. Karazag, B. Coskun, S. Karabey

**Affiliations:** ^1^Psychiatry, İstanbul University Medical Faculty; ^2^Public Health, Bahçeşehir University School of Medicine; ^3^Public Health, İstanbul University Institute of Health Sciences; ^4^Public Health Nursing, Istanbul University - Cerrahpaşa Florence Nightingale Faculty of Nursing; ^5^Bahçeşehir University School of Medicine, İstanbul; ^6^Psychiatry, Kocaeli University Medical Faculty, Kocaeli; ^7^Public Health, İstanbul University Medical Faculty, İstanbul, Türkiye

## Abstract

**Introduction:**

“Community Mental Health (CMH)” is defined as policies and practices aimed at improving mental health of communities and promoting healthy societies. Community mental health issues require a multidisciplinary approach because of their complex pattern, in which social determinants play a direct role in both their causes and solutions. However, training of healthcare professionals on CMH issues is still inadequate in many countries.

**Objectives:**

The purpose of this study was to assess mental health and public health professionals’ awareness on CMH, as well as their opinion on the quality of their postgraduate education on CMH in Türkiye. The findings of this study are expected to provide guidance for the improvement of postgraduate education programs of psychiatry and public health.

**Methods:**

The descriptive quantitative study was conducted with psychiatrists, public health physicians, and nurses with a postgraduate degree in Public Health or Psychiatric Nursing, who voluntarily participated by completing an online questionnaire. Data from a total of 131 physicians (43.5%) and nurses (56.5%) were analyzed by using the SPSS statistical package, where descriptive statistics, chi-square, t-test, and ANOVA were used.

**Results:**

The majority (65.6%) of participating physicians and nurses were employed in tertiary healthcare institutions, with the remainder working in other healthcare settings. While half of the healthcare professionals had CMH topics embedded in their postgraduate education curriculum, only 40% had practical training in Community Mental Health Centers. Only one third (37.4%) expressed confidence in their knowledge of CMH, while only one participant reported feeling adequately informed about CMH services at the central and provincial level in Türkiye. One-third of the participants suggested CMH to become a subspecialty for health professionals, emphasizing the need for dedicated theoretical and practical courses in postgraduate curricula of public health and psychiatry education. The study also highlighted a significant difference between nurses and physicians regarding their postgraduate curriculum and perceived knowledge on CMH.

**Conclusions:**

The study revealed that postgraduate education on CMH is still limited in Türkiye, with more emphasis of CMH in psychiatric nursing education. The findings indicate that education programs need to be revised to include more practical training, including practicum in Community Mental Health Centers and that CMH can potentially become a subspecialty for public health professionals and psychiatrists. Conducting more comprehensive quantitative and qualitative studies on this subject, and enriching existing postgraduate education programs in terms of Community Mental Health, are of great importance for protecting and promoting community mental health.

**Disclosure of Interest:**

None Declared

